# The adoption of assistive smart home technology and health-related quality of life among older adults: the moderating role of depressive symptoms

**DOI:** 10.3389/fpubh.2025.1726983

**Published:** 2025-12-15

**Authors:** Yuwei Qiao, Longtao Wang, Yanli Liu, Weichao Chen

**Affiliations:** School of Journalism and Communication, Hunan Normal University, Changsha, China

**Keywords:** assistive smart home technology, older adults, health-related quality of life (HRQoL), depressive symptoms, aging in place

## Abstract

**Objectives:**

This study aims to assess the impact of assistive smart home technology use on the health-related quality of life (HRQoL) and to examine the moderating effect of depressive symptoms.

**Methods:**

Using data from the 2021 Psychology and Behavior Investigation of Chinese Residents (PBICR), a total of 1,147 participants (aged ≥ 60 years) were included after excluding samples with missing core values. An Ordinary Least Squares (OLS) regression model was employed to examine the association between assistive smart home technology use and HRQoL among older adults.

**Results:**

Assistive smart home technology use was significantly and positively associated with HRQoL among older adults. Moderation analysis further revealed that depressive symptoms strengthened this association, with individuals experiencing higher depressive symptoms showing a greater increase in HRQoL when using assistive smart home technology.

**Conclusion:**

The findings suggest that assistive smart home technology may contribute to improving HRQoL among older adults, particularly for those with higher depressive symptoms. Promoting age-friendly smart home devices, community-based training, and integration with healthcare services may enhance the benefits of technology adoption and support aging in place in China.

## 1 Introduction

The aging of the population has spread throughout the world. By 2019, there will be 1 billion older adults, and by 2050, there will be 2.1 billion (1). Data from the National Bureau of Statistics of China show that by the end of 2024, the number of people aged 60 and above had increased to 310.31 million, representing 22.0% of the total population (2), indicating a further deepening of population aging in China. This demographic shift poses considerable challenges while simultaneously creating opportunities for innovative approaches to older adult care.

Smart home technology (SHT) is rapidly becoming a permanent fixture in our everyday lives (3). The definition of the “smart home” used in this paper is from Demiris and Hensel which is: “a residence wired with technology features that monitor the well-being and activities of their residents to improve overall quality of life, increase independence and prevent emergencies” (4). Smart homes refer to living environments that utilize the sensors, the Internet of Things (IoT), artificial intelligence (AI), and other technologies to facilitate automated home control, energy management, security monitoring (5). SHTs could improve various aspects of daily living for older adults, including safety, health monitoring, and social engagement (6). In the context of older adult care, health-related quality of life (HRQoL) has emerged as a crucial metric. HRQoL encompasses an individual’s health status in physical, psychological, social, and economic aspects. It is widely recognized as an important indicator for assessing the health management for older people (7).

While age-related declines in physical and cognitive function often make professional care necessary, advances in smart home technology offer a promising alternative that enables older adults to maintain effective self-care at home. Research has shown that smart homes are associated with improved quality of life, greater independence, and a reduced risk of emergencies through the use of activity-monitoring systems (8). Supported by the Internet of Things (IoT), these systems integrate sensors and communication technologies for automatic control and remote monitoring, providing safer and more comfortable living conditions for older adults (9).

Despite growing interest in smart home technologies for older adult care, empirical research examining the relationship between assistive SHT use and health-related quality of life (HRQoL) among older adults in China remains limited. Therefore, the present study draws on data from the Psychology and Behavior Investigation of Chinese Residents (PBICR) to investigate the association between assistive SHT use and HRQoL among older adults in China. Specifically, the study examines the moderating role of depressive symptoms. Additionally, the study analyzes heterogeneity across demographic subgroups, including different age, urban–rural residence, and educational levels. By identifying vulnerable populations who may benefit most from smart home interventions, this research aims to provide both theoretical insights and practical guidance for promoting healthy aging and optimizing the implementation of smart home technologies in older adult care settings.

## 2 Literature review

### 2.1 Effect of assistive SHT use on HRQoL of the older adults

It has long been questionable whether smart homes can have a positive impact on health outcomes for older adults. Health-related benefits of smart home included health monitoring and disease management (10), improved access to healthcare services, and promotion of exercise and healthy lifestyles (11). Functional benefits have also been identified, such as supporting older adults with limited mobility. Moreover, smart home technology has been shown to boost older adults’ quality of life by fostering a sense of accomplishment and future security (12). Furthermore, a study has shown that devices like automatically adjusted and remotely controlled lighting systems enhance the convenience and efficiency of daily activities (13).

### 2.2 The moderating effect of depressive symptoms

Depression is defined and understood as a multidimensional condition, involving various aspects such as biology, psychology, and sociology. According to Marikyan (14), the acceptance of smart home technologies by older adults was influenced by their cognitive ability and mental health. Smart home technology could support the aging population, vulnerable people and people with chronic conditions both inside and outside of the house (15–17). Studies suggested that smart homes could improve social interaction and even help users overcome feelings of isolation, potentially contributing to better mental health and reducing the risk of depression (18–20). A recent study found that older adults who used smart bracelets showed significant improvements in several key health indicators such as self-rated health, depression risk, social adaptability and life satisfaction (21). Meanwhile, older adults experiencing depressive symptoms often exhibited poorer emotional and physical health, which could contribute to a lower HRQoL (22).

## 3 Materials and methods

### 3.1 Study population

The data for this study were derived from the Psychology and Behavior Investigation of Chinese Residents (PBICR) (23), conducted from 10 July 2021 to 15 September 2021. This study utilized a multi-stage sampling procedure. (a) 23 provinces, 5 autonomous regions (including Xinjiang, Inner Mongolia, Tibet, and Guangxi), and 4 municipalities directly under the central government (Beijing, Tianjin, Shanghai, and Chongqing) were included. Within each province and autonomous region, 2 to 6 cities were randomly selected, totaling 120 cities. (b) Surveyors or survey teams, each comprising 10 or fewer members, were publicly recruited in these cities. Utilizing data from the “7th National Population Census in 2021,” quota sampling was conducted to ensure that the sample’s gender, age, and urban–rural distribution were representative of the population characteristics. At least one surveyor or survey team was assigned to each city.

Individuals were retained if he or she (a) Age ≥12 years; (b) Citizenship of the People’s Republic of China; (c) Permanent resident of China (annual departure time ≤1 month); (d) Voluntary participation with informed consent; (e) Ability to complete the online questionnaire independently or with the help of a surveyor; (f) Understanding the meaning of each item in the questionnaire. Individuals with any of the following criteria were excluded: (a) Individuals who are confused, mentally abnormal, or have cognitive impairment; (b) Individuals participating in other similar studies; (c) Individuals unwilling to participate in this study.

Surveyors distributed questionnaires one-on-one to the public in their designated areas via the online platform.^1^ Ultimately, the study obtained 11,031 valid questionnaires characterized by high quality, accurate national representation. After excluding missing and invalid variables and respondents aged below 60 years, a final sample of 1,147 was obtained for analysis. The participation in this survey in all respondents was voluntary.

### 3.2 Variables and measurements

#### 3.2.1 Health-related quality of life

The dependent variable, HRQoL, comprises both the utility index derived from the 5-level EQ-5D version (EQ-5D-5L) and the EQ visual analog scale (EQ-VAS) score. The EQ-5D-5L, employed for evaluating the HRQoL among older adults, combines a 5-dimensional health description system with self-reported health status using the EuroQol Visual Analogue Scale (EQ VAS) (24). The health descriptive system comprises mobility, self-care, usual activities, pain/discomfort, and anxiety/depression (25). Each item was rated on a five-point response scale, with 5 indicating ‘extreme problems’, 4 indicating ‘severe’, 3 indicating ‘moderate’, 2 indicating ‘slight’, and 1 indicating ‘no problems’. The Cronbach’s *α* value for HRQoL was 0.798 in this study. The percentage of respondents who reported any problem (scoring 2 to 5) in each domain was calculated. The utility index (UI) score for each respondent was generated using the population preference value sets developed by Yao (26), which range from −0.162 (worst) to 1 (best).

The EQ VAS score mirrors participants’ self-reported overall health perceptions. Responses on the scale measure participants’ perceived health status on a vertical scale of 0 to 100, spanning from “the worst health” to “the best health.” The feasibility, validity, and reliability of the EQ-5D-Y have been documented (27).

#### 3.2.2 Assistive SHT use

This study measures the independent variable using the question “Do you or your home currently have the following smart homes,” with responses coded as 1 = yes and 0 = no. Assistive SHT includes smart clothes hanger, electric curtain, smart air conditioner, smart washing machine, and smart door lock. The assistive SHT score is calculated as the sum of all individual device scores, yielding a minimum value of 1 and a maximum value of 5.

#### 3.2.3 Depressive symptoms

To evaluate the level of depressive symptoms among the older adults, the Patient Health Questionnaire (PHQ-9) was utilized due to its efficacy in assessing depression disorders (28). The PHQ-9 scale was developed by Columbia University in the mid-1990s and is a self-assessment scale specifically designed to screen for mental disorders in primary health care settings (29). The Chinese version of PHQ-9 has been well-validated in multiple studies (30, 31). The PHQ-9 consists of 9 items designed to measure the severity of depressive symptoms. Responses are scored from 0 to 3 (0 = Never, 1 = Several Days, 2 = More Than Half the Days, and 3 = Nearly Every Day). The total score ranges from 0 to 27, with 5, 10, 15, and 20 representing cut points for mild, moderate, moderately severe, and severe depression, respectively (32). Higher scores on the PHQ-9 indicate more severe depressive symptoms. The Cronbach’s alpha coefficient for the PHQ-9 in this study was calculated to be 0.93, indicating good reliability.

#### 3.2.4 Control variables

The control variables selected in this study primarily include age, household monthly income per capita, BMI, gender, household status, educational level, marital status, number of children, insurance status, smoking status, drinking status, chronic diseases status, and disability status. Age was categorized into the nine groups: 60–65 years, 66–70 years, 71–75 years 76–80 years, 81–85 years, 86–90 years, 91–95 years, 96–100 years, and 101 years or older, with assigned values from 1 to 9, respectively. Household monthly income per capita was categorized into eleven groups: ≤1,500, 1,501–3,000, 3,001–4,500, 4,501–6,000, 6,001–7,500, 7,501–9,000, 9,001–10,500, 10,501–12,000, 12,001–13,500, 13,501–15,000, and ≥15,001, with assigned values from 1 to 11, respectively.

### 3.3 Statistical analysis

First, in the descriptive analysis of respondents’ baseline characteristics, percentages were used for binary or categorical variables, while mean ± standard deviation (SD) was used for continuous variables. Second, the Ordinary Least Squares (OLS) regression model was initially employed to investigate the potential the potential association between assistive SHT use and HRQoL among older adults. Meanwhile, this study utilized propensity score matching (PSM) as a methodology to address potential endogeneity issues. Third, a moderation model was conducted to test whether depressive symptoms moderated this association.

The data were analyzed using R version 4.4.1 (R Foundation for Statistical Computing, Vienna, Austria).

## 4 Results

### 4.1 Descriptive analysis

Table 1 presents the descriptive statistics of the variables. Participants who had used any of these devices were assigned a value of 1, whereas those who had not were assigned a value of 0. Among the 1,147 participants, 495 used assistive SHT, while 652 did not. The EQ-5D index scores were similar between users and non-users.

**Table 1:** Characteristics of participants included in the study.

Variable	Assistive SHT use		*p*
	No (*N* = 652)	Yes (*N* = 495)	
Continuous variables			
EQ-5D index	0.9 ± 0.2	0.9 ± 0.2	0.021
EQ VAS score	72.7 ± 19.8	75.5 ± 19.3	0.019
Age			0.071
60–70	273(41.9%)	241(48.7%)	
71–80	314(48.2%)	211(42.6%)	
≥81	65(10%)	43(8.7%)	
Household monthly income per capita	3.2 ± 2.2	3.7 ± 2.2	<0.001
BMI, kg/m^2^, Mean (SD)	22.1 ± 3.3	22.2 ± 3.1	0.388
Categorical variables			
Gender (Male)	345 (52.9%)	236 (47.7%)	0.090
Household status (Urban)	270 (41.4%)	259 (52.3%)	<0.001
Educational level			<0.001
Uneducated	141 (21.6%)	68 (13.7%)	
Primary school	181 (27.8%)	116 (23.4%)	
Secondary school	164 (25.2%)	129 (26.1%)	
High school	66 (10.1%)	72 (14.5%)	
College (Associate)	40 (6.1%)	40 (8.1%)	
College (Undergraduate)	51 (7.8%)	53 (10.7%)	
Master’s degree	1 (0.2%)	8 (1.6%)	
Doctoral degree	8 (1.2%)	9 (1.8%)	
Marital status (Married)	505 (77.5%)	389 (78.6%)	0.699
Number of children			0.326
No children	37 (5.7%)	28 (5.7%)	
One child	157 (24.1%)	135 (27.3%)	
Two children	224 (34.4%)	179 (36.2%)	
Three children	234 (35.9%)	153 (30.9%)	
Insurance (Yes)	578 (88.7%)	439 (88.7%)	1.000
Non-smoker (Yes)	428 (65.6%)	325 (65.7%)	1.000
Non-drinker (Yes)	453 (69.5%)	328 (66.3%)	0.274
Chronic diseases (Yes)	416 (63.8%)	307 (62%)	0.577
Disability (Yes)	68 (10.4%)	64 (12.9%)	0.222

Assistive SHT users were more likely to live in urban areas (52.3% vs. 41.4%). Users had a lower proportion of males (47.7%) compared with non-users (52.9%). Educational attainment was generally higher among users, with more participants having completed secondary school or higher education levels. Marital status revealed a slightly higher percentage of married individuals among users (78.6% vs. 77.5%). Lifestyle choices, such as non-smoking and non-drinking, were similarly distributed between users and non-users. Additionally, there was no significant difference in the prevalence of chronic diseases and disabilities between the two groups.

Overall, this analysis suggests that the use of assistive SHT among the older adults is associated with better HRQoL, higher household income, greater urban residency, and higher educational attainment.

### 4.2 Benchmark regression

Table 2 presents the results of a linear regression analysis that examines the association between assistive SHT use and HRQoL among older adults. The analysis differentiates between the EQ-5D index and EQ-VAS score as measures of HRQoL. Models 1 and 3 encompass only the independent variable, specifically the use of assistive SHT, while Models 2 and 4 integrate both the principal explanatory variable and a comprehensive array of control variables.

**Table 2:** Linear regression of assistive SHT use on HRQoL. (*N* = 1,147).

Variables	EQ-5D index				EQ VAS score			
	Model 1		Model 2		Model 3		Model 4	
	β(SE)	95% CI	*β* (SE)	95% CI	*β* (SE)	95% CI	*β* (SE)	95% CI
Assistive SHT use	0.021^***^ (0.006)	(0.009,0.031)	0.016^***^ (0.005)	(0.067,0.028)	2.232^***^ (0.608)	(1.038, 3.424)	1.690^***^ (0.592)	(0.568, 2.895)
Gender			0.033^**^ (0.013)	(0.008,0.059)			2.82^**^ (1.408)	(0.200, 5.730)
	Age (ref = 60–70)												
71–80			−0.007 (0.011)	(−0.029, 0.014)				−0.222 (1.201)		(−2.577, 2.132)			
≥81			−0.056^**^ (0.019)	(−0.095, −0.019)			0.937 (2.089)	(−3.162, 5.037)
Household status			−0.002 (0.011)	(−0.025, 0.020)			0.692 (0.240)	(−1.741, 3.126)
Educational level					0.008 (0.008)	(−0.008, 0.024)					1.590 (0.874)	(−0.126, 3.306)
Marital status			0.034** (0.012)	(0.009, 0.059)					2.726* (1.370)	(0.037, 5.416)		
Household monthly income per capita					0.004 (0.003)	(−0.001, 0.010)					0.198 (0.291)	(−0.374, 0.771)
Number of children			0.002 (0.006)	(−0.009, 0.015)					−0.685 (0.675)	(−2.011, 0.640)		
Insurance					0.031^*^ (0.016)	(0.001, 0.063)					5.760*** (1.739)	(2.347, 9.174)
BMI			0.001 (0.002)	(−0.001, 0.005)					0.306 (0.175)	(−0.039, 0.652)		
Non-smoker					0.004 (0.013)	(−0.022, 0.031)					2.592 (1.469)	(−0.291, 5.476)
Non-drinker			−0.002 (0.012)	(−0.026, 0.021)					−0.632 (1.303)	(−3.201, 1.935)		
Chronic diseases					−0.086^***^ (0.011)	(−0.108, −0.065)					−8.339^***^ (1.175)	(−10.654, −6.025)
Disability			−0.138^***^ (0.016)	(−0.171, −0.106)					−7.178^***^ (1.784)	(−10.686, −3.671)		
*N*	1,147				1,147		1,147				1,147	
*R* ^2^	0.014		0.175		0.015		0.114	

*p < 0.05; **p < 0.01; ***p < 0.001.

The results indicate that the adoption of assistive SHT is significantly positively associated with the HRQoL of older adults. For the EQ-5D index, Model 1 shows a significant positive association, which remains significant in Model 2 after including control variables. Similarly, for the EQ-VAS score, Model 3 indicates a significant positive association, which also persists in Model 4 with control variables.

Across Models 2 and 4, gender, marital status, and insurance exhibit a significant positive correlation with EQ-5D index and EQ-VAS score. Chronic diseases and disability exhibit a significant negative correlation with HRQoL. Specifically, older adults who are male, married, possess insurance, and do not suffer from chronic diseases or disabilities tend to have higher HRQoL.

### 4.3 Results of robustness analysis

To address the prevalent issue of selection bias in social science research, this study employed the propensity score matching (PSM) method. Assistive SHT users were divided into treatment and control groups to simulate random assignment. This approach ensured no significant differences in the observed covariates between the matched treated and control groups.

The K-nearest neighbor matching method was employed based on audiobook usage status in the sample. As shown in Table 3, the matched variables exhibited significantly reduced skewness compared to pre-matching, with the majority of standard deviations falling below 6%. After matching, the means of the treatment and control groups were closer, and there were no significant differences between the two groups, indicating that sample selection bias had been largely eliminated.

**Table 3:** Results of balance test.

Variable		Mean		Deviation rate (%)	*t*-test	
		Treatment group	Control group		*t*-value	*p* > |*t*|
Gender	Unmatched	0.476	0.529	−10.500	−1.760	0.079
	Matched	0.476	0.475	0.200	0.040	0.970
Household status	Unmatched	0.523	0.414	22.000	3.690	0.000
	Matched	0.523	0.531	−1.700	−0.270	0.785
Marital status	Unmatched	0.785	0.774	2.700	0.460	0.647
	Matched	0.785	0.794	−2.100	−0.340	0.732
Educational level	Unmatched	9.147	7.627	28.800	4.840	0.000
	Matched	9.147	8.867	5.300	0.840	0.398
Household monthly income per capita	Unmatched	3.656	3.210	20.300	3.410	0.001
	Matched	3.656	3.686	−1.400	−0.210	0.836
Number of children	Unmatched	1.923	2.004	−9.00	−1.510	0.131
	Matched	1.923	1.954	−3.500	−0.540	0.588
Insurance	Unmatched	0.886	0.886	0.100	0.020	0.985
	Matched	0.886	0.871	4.900	0.750	0.456
BMI	Unmatched	22.215	22.052	5.200	0.860	0.388
	Matched	22.215	22.269	−1.700	−0.270	0.790
Non-smoker	Unmatched	0.656	0.656	0.000	0.000	0.997
	Matched	0.656	0.635	4.400	0.690	0.492
Non-drinker	Unmatched	0.662	0.694	−6.900	−1.160	0.248
	Matched	0.662	0.665	−0.600	−0.090	0.925
Chronic diseases	Unmatched	0.620	0.638	−3.700	−0.620	0.536
	Matched	0.620	0.623	−0.600	−0.090	0.927
Disability	Unmatched	0.1292	0.104	7.800	1.310	0.189
	Matched	0.1292	0.128	0.200	0.020	0.981

The results in the table were obtained using the k-nearest neighbor matching method with calipers.

The study will confirm that the use of assistive SHT by older adults is a self-selected behavior rather than a random selection through robustness tests using propensity score matching (Table 4). The results of k-nearest neighbor matching, nearest neighbor matching, and radius matching all indicate that assistive SHT use by older adults has a significant impact on their HRQoL, and the benchmark model results are robust.

**Table 4:** The average treatment effect of assistive SHT use on HRQoL.

Variables	Matching method	Treatment group (1)	Control group (2)	ATT value	Standard deviation	*t*-value
EQ-5D index	Before the match ATT	0.890	0.864	0.025	0.011	2.31**
	After the match ATT					
	K-nearest neighbors matching	0.890	0.868	0.021	0.012	1.69*
	Nearest neighbor matching	0.890	0.865	0.024	0.010	2.25**
	Radius matching	0.889	0.869	0.020	0.011	1.75*
EQ VAS score	Before the match ATT	75.456	72.716	2.740	1.166	2.35**
	After the match ATT					
	K-nearest neighbors matching	75.456	73.537	1.919	1.349	1.69*
	Nearest neighbor matching	75.456	73.146	2.310	1.349	2.01**
	Radius matching	75.219	73.053	2.166	1.221	1.77*

*p < 0.05; **p < 0.01.

### 4.4 Results of heterogeneity analysis

This study conducts a heterogeneity analysis along three dimensions: age, household registration status, and educational attainment, to elucidate how assistive SHT use influences the HRQoL of older adults across different subgroups. The results of the individual heterogeneity analysis are detailed in Table 5.

**Table 5:** Heterogeneity analysis.

Variable	Age		Household status		Educational level		
	60–75	>76	Rural	Urban	Primary school and below	Secondary or high school	College or above
EQ-5D index	0.013* (0.005)	0.032* (0.017)	0.019* (0.008)	0.012 (0.007)	0.034** (0.010)	0.001 (0.008)	0.018 (0.009)
*R* ^2^	0.161	0.195	0.232	0.158	0.220	0.120	0.195
EQ VAS score	1.647* (0.660)	2.264 (1.385)	2.362* (0.929)	1.207 (0.762)	1.709 (1.048)	2.025* (0.928)	1.265 (1.217)
*R* ^2^	0.113	0.195	0.138	0.123	0.160	0.091	0.057
Control variables	Y	Y	Y	Y	Y	Y	Y
*N*	875	272	618	529	506	431	210

*p < 0.05; **p < 0.01. The parentheses are standard errors.

Compared to older adults aged 60–75, the higher EQ-5D index associated with the utilization of assistive SHT was notably more pronounced for those aged over 76. For the EQ-VAS score, assistive SHT use shows a significant positive effect for the 60–75 age group, but the effect is not significant for those aged over 76. Regarding household registration status, assistive SHT use is significantly positively associated with the EQ-5D index and EQ-VAS score among rural residents (correlation coefficient = 0.019), but no such association is observed for urban residents. When examining educational attainment, assistive SHT use is significantly positively associated with the EQ-5D index among individuals with a primary school education or below, but no such association is observed for other education levels. For the EQ-VAS score, it shows a significant positive association only among those with secondary or high school education.

### 4.5 Moderating effect of depressive symptoms

The test of the moderating effect is based on the method proposed by Wen (33). The interaction term between assistive SHT use and depressive symptoms is significant for both outcomes. As shown in Table 6, the interaction terms for both EQ-5D index (b = 0.002, *p* < 0.01) and EQ VAS score (b = 0.256, *p* < 0.01) are positive. This suggests that the beneficial association between assistive SHT use and HRQoL is significantly stronger among individuals with higher levels of depressive symptoms.

**Table 6:** The results of the moderation effect analysis.

Variable	EQ-5D index		EQ VAS score	
	Model 5	Model 6	Model 7	Model 8
Assistive SHT use	0.017** (0.005)	0.020*** (0.005)	1.732** (0.592)	1.993*** (0.565)
Depressive symptoms		−0.014*** (0.001)		−1.200*** (0.105)
Assistive SHT use × Depressive symptoms		0.002** (0.001)		0.254** (0.091)
Control variables	Yes	Yes	Yes	Yes
*N*	1,147	1,147	1,147	1,147
*R* ^2^	0.085	0.247	0.113	0.204

**p < 0.01; ***p < 0.001.

**Figure 1:**
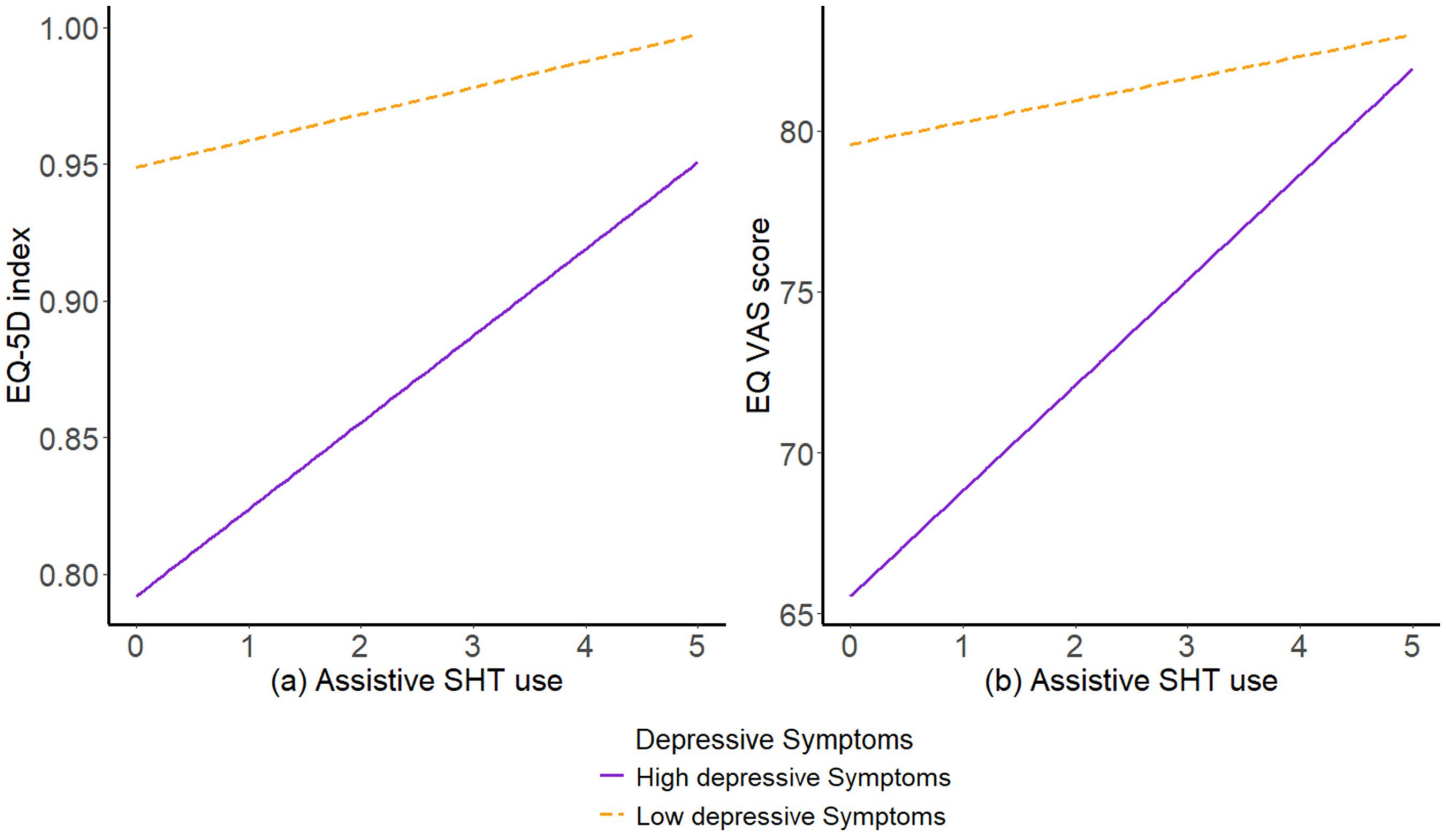
Interactions of assistive SHT use with depressive symptoms in relation to HRQoL.

To further explore the association between assistive SHT use and HRQoL, simple slopes are tested at low (M-1SD), moderate (M), and high (M + 1SD) levels of depressive symptoms (Figure 1). As shown in both subgraphs (a) and (b) of Figure 1, assistive SHT use was significantly associated with higher EQ-5D index and EQ VAS scores among participants with high depressive symptoms, represented by the steeper positive slope of the purple lines. However, no significant relationship was found between smart home use and these HRQoL outcomes for participants with low depressive symptoms.

## 5 Discussion

This study reveals that assistive SHT use show a significant positive impact on HRQoL among older adults in China. This finding is consistent with previous research suggesting that smart home technologies can enhance independence, safety, and well-being in older populations (34, 35). This finding aligns with previous research, which suggests that smart home technologies (e.g., video doorbells, light sensors and smart locks) support home security (36), thereby strengthening the HRQoL of older adults (37, 38). SHT enable older adults to successfully complete instrumental activities of daily living (IADLs) that they might otherwise struggle with due to age-related functional decline (39).

Compared with those aged 60–75, assistive SHT use is more likely to be associated with a higher EQ-5D index among individuals over 76, highlighting the potential of these technologies to support aging in place in later life. Assistive SHT use shows positive associations with EQ-VAS only among participants aged 60–75, whereas no significant association is observed among individuals over 76. Assistive SHT use shows positive associations with the EQ-5D index and EQ-VAS only among rural participants, whereas no significant associations are observed among urban individuals. This may be because assistive SHTs are more common in urban areas, which could help explain the absence of significant effects there. To enhance the benefits for urban older adults, providing higher-quality smart home devices may be necessary.

Furthermore, the finding that depressive symptoms moderate the relationship between assistive SHT use and HRQoL, with those experiencing higher levels of depressive symptoms showing more significant improvements in HRQoL from assistive SHT use, is particularly noteworthy. This is consistent with prior research showing that technology-based or assistive smart home use tend to yield greater mental health and quality-of-life benefits among older adults with elevated depressive symptoms. Lee et al. (40) found that socially assistive robot use significantly reduced depressive symptoms and improved HRQoL among socially isolated older adults. Yen et al. (41) similarly reported that social robot interventions produced substantial reductions in depression and loneliness among older adults, with particularly strong effects observed in those with higher baseline depressive symptoms. This suggests that smart home technologies may have a more profound impact on those struggling with mental health issues, potentially offering a novel approach to supporting older adults with depressive symptoms.

Based on the findings discussed, here are some recommendations for promoting and optimizing the use of smart home devices among older adults:

Firstly, to promote SHT use among older adults, it is crucial to develop and implement comprehensive community-level training initiatives. These initiatives could enhance older adults’ willingness to use SHT and improve their digital literacy by focusing on addressing common concerns and highlighting the benefits of smart home technologies for daily living and health management. Simultaneously, manufacturers should modify SHT to better suit older users by incorporating simplified interfaces, larger buttons, voice-activated controls (42), and easy-to-read displays. Liu (43) found that older adults’ satisfaction with smart home technology improves when assistive devices are personalized to their needs, support their usual activities, and are relatively easy to use.

Secondly, to tailor SHT use for specific groups, focus on increasing accessibility and awareness in rural areas by partnering with local governments and community organizations. These partnerships can help subsidize devices, provide installation support, and offer ongoing technical assistance. Given that the study indicates individuals with higher depressive symptoms experience greater HRQoL improvements from SHT use, it is important to collaborate with hospitals, psychological clinics, and other healthcare institutions to integrate SHT into auxiliary treatment plans for older adults with depression. Furthermore, as suggested by Ghorayeb (44), enhancing SHT with features such as entertainment functions, online shopping, secure social networking, and family connectivity may increase their appeal to older adults. These enhancements can help alleviate depressive symptoms by keeping users connected with loved ones and engaged in enjoyable activities, thereby reducing social isolation and improving overall quality of life.

While this study provides valuable insights, several limitations should be noted. First, the analysis focused solely on assistive SHT, excluding other types of smart home technologies such as entertainment and health-related devices. Future research could include a broader range of smart home technologies to provide a more comprehensive understanding of their impacts. Second, the higher HRQoL among urban older adults may reflect contextual resource disparities rather than individual health status alone, particularly given urban advantages in healthcare, digital infrastructure, and smart home access, as well as potential sample imbalances. Third, this study measures assistive SHT as a simple sum of devices (range 1–5), which assumes equal weight or impact of each device on HRQoL. In reality, different devices may exert varying effects depending on individual needs and contextual factors. Future research should examine device-specific impacts or adopt weighted scoring approaches. Finally, unobserved factors such as personal attitudes, family support, or prior health behaviors may influence both HRQoL and smart home adoption, and reverse causality cannot be ruled out. Future research should use stratified sampling and control for structural resource inequalities.

## 6 Conclusion

This study confirms that assistive SHT use is positively associated with HRQoL among older adults in China. Depressive symptoms moderate this relationship, showing stronger positive effects for individuals with higher levels of depressive symptoms.

These findings suggest important implications for policymakers and healthcare providers. To enhance HRQoL and support healthy aging, efforts should focus on increasing access to and adoption of SHT, particularly for vulnerable subgroups such as rural residents and those with depressive symptoms. The study highlights the potential of SHT to support aging in place, especially for older adults in rural areas. Specifically, tailored community-based training programs and age-friendly device adaptations can improve older adults’ digital literacy and willingness to use these technologies. Additionally, integrating smart home devices into treatment plans for depression could offer a novel approach to mental health support.

## Data Availability

Publicly available datasets were analyzed in this study (https://www.x-mol.com/groups/pbicr). Data are available upon reasonable request by emailing Yibo Wu at: bjmuwuyibo@outlook.com.

## Footnotes

1
https://www.wjx.cn/
